# Characterization of Immobilized Magnetic Fe_3_O_4_ Nanoparticles on *Raoultella Ornithinolytica* sp. and Its Application for Azo Dye Removal

**DOI:** 10.1007/s12010-022-04076-3

**Published:** 2022-07-26

**Authors:** Fatma Bekhit, Soha Farag, Ahmed M. Attia

**Affiliations:** 1 Department of Environmental Studies, Institute of Graduate Studies and Research, Alexandria University, Alexandria, Egypt; 2 Environmental Biotechnology Department, Genetic Engineering and Biotechnology Research Institute, City of Scientific Research and Technological Applications, Alexandria, Egypt

**Keywords:** Magnetite nanoparticles, *Raoultella Ornithinolytica* sp*.*, Immobilization, Dye removal, Continuous fed batch, Toxicity test

## Abstract

A high-performance immobilized bacterial strain coated with magnetic iron oxide nanoparticles was used for Basic Blue 41 azo dye (BB 41 dye) decolorization. To create the coated bacterial strain, *Raoultella Ornithinolytica* sp. was isolated and identified under the accession number KT213695, then coated with manufactured magnetic iron oxide nanoparticles. SEM and SEM–EDX were used to characterize the coated bacteria and validate its morphological structure formation. The coated *Raoultella Ornithinolytica* sp. A1 (coated A1) generated a 95.20% decolorization for BB 41 dye at 1600 ppm starting concentration with an optimal dose of coated A1 5 mL/L, pH 8, under static conditions for 24 h at 37 °C. Continuous batch cycles were used, with BB 41 dye (1600 ppm) added every 24 h four times, to achieve a high decolorization efficiency of 80.14%. Furthermore, the metabolites of BB 41 dye biodegradation were investigated by gas chromatographic-mass spectrum analysis (GC–MS) and showed a less toxic effect on the bioindicator *Artemia salina*. Additionally, 5 mL/L of coated A1 demonstrated the highest decolorization rate (47.2%) when applied to a real wastewater sample after 96 h with a consequent reduction in COD from 592 to 494 ppm.

## Introduction

Water pollution is still a major global environmental issue; the United Nations estimates that unsafe or insufficient access to water causes around 3.1% of fatalities worldwide or over 1.7 million deaths each year. Wistfully, pollution of several water sources increases environmental hazards to creatures and other living beings. Therefore, the most effective techniques of safeguarding the rare freshwater resources have been identified as saving the usage of water resources and reutilization of treated wastewater for various uses [1, 2].

Dyes have been used by humans for over a thousand years for a variety of purposes; natural dyes were made on a modest scale using naturally available materials such as plants or insects. Natural dyes, unfortunately, had a limited range of colors, and they faded when exposed to sunshine or washed. Because of the increasing need for dyes, synthetic dyes were found and large-scale production began. The problems of natural dyes, which are widely utilized in industry, were solved by the invention of synthetic dyes, which are used to provide numerous stable color, not fade when exposed to light, sweat, water, or moisture [3, 4].

Around 10,000 different dyes are generated globally, and the textile industry consumes approximately 8 × 10^5^ t of synthetic dyes. According to World Bank estimates, textile dyeing and finishing treatments contribute with 17 to 20% of industrial wastewater contamination [5, 6]. Azo dyes are the most prevalent due to their inexpensive cost, simplicity of manufacture, stability, and great variety of colors available. Azo dyes are known to have a negative impact on chemical oxygen demand (COD), biological oxygen demand (BOD), dissolved salts (TDS and TSS), as well as dangerous compounds such as heavy metals. The metabolites of several synthetic azo dyes also have cancerous, pathogenic, and lethal effects [7, 8].

The elimination of dyes from the effluent wastewater is a difficult task that can be developed using several physical, chemical, and biological methods. Physical techniques (direct methods), include adsorption, flocculation or coagulation, membrane filtration, irradiation, ion exchange, nano-filtration or ultra-filtration, and reverse osmosis. These strategies are frequently used because they are simple and effective. However, challenges with its restoration or removal, as well as significant sludge output, have made this strategy undesirable [9]. Chemical procedures for dye removal include advanced oxidation, photochemical, electrochemical destruction, ozonation, Fenton reaction, and ultraviolet irradiation. Chemical dye removal procedures are unappealing because they are expensive, require specialized equipment, and use a lot of electricity in comparison to other dye removing techniques [10, 11].

Alternatively, biological treatment of azo dyes is favored since it can degrade practically all dyestuffs and also overcomes many of the physicochemical procedures’ drawbacks [12]. For dye-containing wastewater treatment, biodegradation is an economical and eco-friendly option [13]. Adsorption on microbial biomass (biosorption) and/or biodegradation of the dyes by various microorganisms such as bacteria, fungi, yeasts, algae, and plants are two methods for decolorization and degradation of azo dyes. Significantly bacteria are the most commonly used microorganisms in synthetic dye decolorization, because they have the ability to accomplish a greater level of mineralization and biodegradation [14, 15].

Nanotechnology has been utilized efficaciously in water/wastewater treatment and emerged as a rapidly upgraded field. Nanoparticles have key properties that make them a good choice as nano adsorbents. It has distinctive characteristics such as minor size, catalytic potential, high reactivity, great surface to volume ratio, high capacity, and selectivity. Additionally, to increase their affinity for target chemicals, nanoparticles can be combined with a variety of chemical groups [16, 17].

Numerous research studies adopted the use of multiple technologies in combination; for example biological treatments paired with nanoparticles’ high adsorption capacity could boost the efficacy of biological treatments [18]. The term “immobilization” refers to the restriction of bacterial cell mobility to a specific space or location. The magnetic immobilization of bacteria has taken on a new dimension attributed to iron oxide nanoparticles (Fe_3_O_4_ NPs). The use of magnetic nanoparticles to decorate bacterial cells and the trapping of bacteria within magnetic nanoparticle clusters are both examples of magnetic immobilization. The immobilized bacterial cells become susceptible to the magnetic field and can then be manipulated by employing this technology. The use of super paramagnetic Fe_3_O_4_ NPs immobilized on bacterial cells with suitable surface chemistry is a novel technology that is most commonly utilized for the magnetic separation of target bacterial cells from a bacterial mixture in samples [19].

The frequently utilized magnetic iron oxide nanoparticles are the most prevalent materials. These magnetic particles offer many advantages, such as low toxicity, good biocompatibility, chemically and mechanically stable during storage, easy to handle, simplicity of cell separation without affecting the stability and recyclability of immobilized cells, resulting in easier downstream treating and safe disposal [20, 21].

The wastewater effluent produced by textile mills and dyeing companies is frequently brightly colored and contains a lot of pollution. Aside from having a poor visual impact on the receiving aquatic habitat, some azo dyes, and biotransformation products are hazardous to aquatic creatures, with some of these substances being carcinogenic and mutagenic. The acute toxicity test, which uses *Artemia salina* as a test organism, is used to examine the success of the treatment process on wastewater by evaluating probable by-products toxicity resulting from biodegradation treatment [22, 23].

The current research focuses on the decolorization and degradation of Basic Blue 41 azo dye, which is used in the textile industry by employing high-performance prepared bacterial strain coated with magnetic iron oxide nanoparticles to improve biological degradation. The structure and composition of coated bacterial cells were investigated. Different factors for dye decolorization were optimized such as dye concentrations, pH, the dosage of coated bacteria, incubation time, temperature, and static or shaking conditions. Furthermore, a toxicity test was studied using *Artemia salina* for the metabolites of BB 41 dye biodegradation. Finally, treatment of real wastewater samples was achieved by using coated bacterial strains.

## Materials and Methods

### Materials

I Basic Blue 41 azo dye (BB 41) was supplied from local textile and dying factors in Alex, Egypt, which was utilized without any additional purification. Ferrous sulfate heptahydrate (FeSO_4_⋅7H_2_O), ferric chloride hexahydrate (FeCl_3_⋅6H_2_O), and sodium hydroxide (NaOH) were purchased from Sigma-Aldrich, besides absolute ethanol (C_2_H_5_OH) (HPLC grade) from Fisher Scientific, UK.
II For the acute toxicity test, dry encysted eggs of *Artemia salina* (Origin: salt lake, USA) were purchased from Sera, Germany.

### Isolation and Purification of Decolorizing Bacterial Isolates

The soil sediment and wastewater samples were gathered from different locations adjacent to the waste of some local textile industrial factories in Alexandria, Egypt. Ten grams of each of the three soil samples or 5 mL of wastewater sample (totally four samples) were inoculated into 100 mL of three different media: LB medium containing (g/L) 10 g peptone, 5 g yeast extract, 5 g sodium chloride (pH 7.0); Zhou and Zimmermann ZZ medium containing (g/L) 5 g yeast extract, 5 g glucose, 0.5 g (NH_4_)_2_SO_4_, 2.66 g KH_2_HPO_4_, 4.32 g Na_2_HPO_4_ (pH 7.0) [24]; and minimal salt (MS) medium containing (g/L) 1.8 g K_2_PO_4_, 1.2 g KH_2_PO_4_, 4.0 g NH_4_Cl, 0.2 g MgSO_4_·7H_2_O, 0.1 g NaCl, 0.01 g FeSO_4_·7H_2_O (pH 7.0) [25]. The medium was mixed with 50 ppm of BB 41 dye, tightly sealed, and incubated at 30 °C for 3 days under shaking conditions (200 rpm). The medium was decolorized, and then 100 μL aliquots of tenfold serially dilution samples were spread onto LB, ZZ, and MSM media agar plates with 50 ppm of BB 41 at 30 °C for 24 h. The morphologically distinct colonies were streaked on agar plates for purification. The three different media (broth and agar plates) were tested. According to the decolorization area shown on the agar plate, the best medium was selected for further experiments. The bacterial isolates with the strongest decolorizing capability assigned a prefix of “A” followed by numbers in a series from 1 to 7 (A1, A2, A3, A4, A5, A6, and A7).

### Decolorization of Azo Dye

The bacterial isolates (from A1 to A7) were selected for further examination and were studied for their decolorization potential on the BB 41 dye. In 250-mL autoclaved Erlenmeyer flasks, 50 mL of sterilized LB broth medium was mixed with 100 ppm of BB 41 and 4% (v/v) inoculums of each isolate separately (the preculture with OD: 1.4 at 600 nm). The flasks were tightly wrapped and incubated for 48 h under shaking conditions at 30 °C/200 rpm. The culture supernatant was used for spectrophotometric analysis at 617 nm (λ max of BB 41) to determine the decolorization percentage of the dye [26].

To determine the most effective isolate for decolorization of the BB 41 dye at different concentrations (100 to 1200 ppm), the dye was mixed with 4% inoculums of uniform cell density (OD: 1.4) at 600 nm for each isolate (A1, A4, and A7) incubated at 30 °C/200 rpm. Decolorization was measured after 48-h incubation at 617 nm using a UV–Vis PG/T60 spectrophotometer. The decolorization percentage was calculated using the following equation, while the distilled water was used as a blank; samples containing BB 41 dye were used as control and each experiment’s culture supernatant was used as a sample [27].

$$\mathrm D\mathrm e\mathrm c\mathrm o\mathrm l\mathrm o\mathrm r\mathrm i\mathrm z\mathrm a\mathrm t\mathrm i\mathrm o\mathrm n\%=\left[\mathrm R\mathrm e\mathrm a\mathrm d\mathrm i\mathrm n\mathrm g\;\mathrm o\mathrm f\;\left(\mathrm C\right)\;\mathrm{decolorization}-\mathrm R\mathrm e\mathrm a\mathrm d\mathrm i\mathrm n\mathrm g\;\mathrm o\mathrm f\;\left(\mathrm S\right)\;\mathrm{decolorization}\right]/\mathrm{Reading}\;\mathrm{of}\;\left(\mathrm C\right)\;\mathrm{decolorization}\times100$$

where C = control, S = sample.

### Molecular Identification of the Bacterial Isolate

In LB medium, a purified colony of the A1 bacterial isolate was cultivated to log phase. Centrifugation was used to obtain the A1 bacteria pallet. Then, by using a bacterial genomic DNA Isolation Kit (RKT09), the genomic DNA was extracted from the isolated bacteria. Amplification was set using prokaryotes 16S rRNA specific forward primer, 5′-AGAGTTTGATCMTGGCTCAG-3′, and reverse primer, 5′-TACGGYACCTTGTTACGACTT-3′. Amplified product was purified using a Qiagen gel extraction kit and the purified PCR products were sequenced. To associate the bacteria isolate, the 16S rRNA sequence (999 bp) was compared to currently known microorganism sequences in GenBank by BLAST (www.ncbi.nlm.nih.gov/blast) sequence analysis. The GenBank accession number for isolate A1 is KT213695 [28].

### Optimization of A1 Growth Conditions for BB 41 Dye Decolorization

This experiment was applied to maximize the ability of the A1 isolate to decolorize BB 41. A1 cells were inoculated and mixed with BB 41 in 250-mL autoclaved Erlenmeyer flasks containing 100 mL of sterile LB medium. The effects of different physiochemical factors on bacterial decolorization were assessed, such as initial dye concentrations (200–3200 ppm), pH (2.0–10.0), temperature (25–45 °C), static or shaking conditions (200 rpm), and incubation time (3–24 h). The removal % was determined by scanning the supernatant in the range of 200 to 700 nm with a UV–Vis spectrophotometer and measuring it at 617 nm.

### Magnetic Iron Oxide Nanoparticles Synthesis

Co-precipitation of Fe^+3^ and Fe^+2^ ions in a 2:1 molar ratio solution with highly basic solutions was used to make magnetic iron oxide nanoparticles. In this procedure, 200 mL of distilled water containing 7.568 g FeCl_3_·6H_2_O and 3.892 g FeSO_4_·7H_2_O was heated to 70 °C, then the alkaline solution of 5 M NaOH (50 mL) was added dropwise with vigorous stirring for 30 min under inert conditions. A magnetic field was used to separate the created black precipitate, and the supernatant was removed by decantation. The precipitate was washed with ethanol and deionized water numerous times. The particles were then dried for 24 h [29, 30].

### Bacterial Cells Coated with Magnetic Iron Oxide Nanoparticle Preparation

Strain A1 was cultivated in 500 mL of LB medium for 24 h at 30 °C with shaking (200 rpm). Centrifugation at 6000 rpm for 15 min was used to collect the precipitated bacteria. To allow the Fe_3_O_4_ NPs and bacterial cell physical contact, 5 g of wet weight bacterial cells were combined with 2.5 g Fe_3_O_4_ NPs in a 1-L culture medium (LB). The flask was incubated for 2 h at 30 °C with 200 rpm agitation to allow Fe_3_O_4_ NP adsorption on the surface of the bacterial cells. An external magnet was used to distinguish the coated bacterium cells from the rest of the mixture. Coated cells were then gently washed twice with distilled water. The biodegradation and decolorization of Basic Blue 41 dye were investigated using immobilized bacterial cells coated with Fe_3_O_4_ NPs (coated A1) [29, 30].

### Magnetic Nanoparticles and Coated Bacterial Cells: Characterization

I The surface and morphology of synthesized Fe_3_O_4_ NPs were scanned with the scanning electron microscope (SEM) (JEOL JSM-5300), transmission electronic microscopy (TEM) (JEOL JSM 6360LA, Japan), and powder X-ray diffraction (XRD) (Shimadzu-7000, USA) to determine the presence of Fe_3_O_4_ NPs nano-crystalline structure according to Bekhit et al. [29].
II The structure and composition of immobilized bacterial cells were investigated utilizing energy-dispersive X-ray spectroscopy (EDX) (EDX-JEOL JSM6360LA) to know the properties of the prepared coated A1 with magnetic Fe_3_O_4_ NPs.

### Optimization of Prepared Coated A1 for BB 41 Dye Decolorization

The purpose of this experiment was to maximize the potential of coated A1 to decolorize BB 41 dye. In 250-mL autoclaved Erlenmeyer flasks containing 100 mL of sterilized LB medium, coated A1 cells were inoculated and BB 41 was added. The flask was tightly sealed and incubated at 30 °C for 24 h. The removal % was determined by scanning the supernatant in the range of 200 to 700 nm with a UV–Vis spectrophotometer and measuring it at 617 nm. The effects of several physiochemical factors, including initial dye concentrations (200–3200 ppm), pH (2.0–10.0), temperature (25–45 °C), static or shaking conditions (200 rpm), coated bacterial cell dosage (0.1–50 mL/L), and incubation period (3–24 h) were all investigated. Each experiment was done independently 3 times and the mean was calculated.

### Continuous Batch Cycle Process

For assessment of BB 41 dye repeated decolorization, the coated A1 with Fe_3_O_4_ NPs was put to the test. The experiment was carried out utilizing the most efficient dose of coated A1 (0.5 mL/100 mL culture media) in combination with the best conditions for BB 41 dye decolorization (LB broth medium pH 8, static conditions at 37 °C, and a 24-h incubation period) and 1600 ppm of BB 41 dye. The coated A1 was removed from the dye solution by an external magnet after the first batch cycle (24 h). In the second batch cycle, the identical concentration of BB 41 dye (1600 ppm) was added and incubated for another 24 h under similar conditions. The third and fourth cycles were carried out in the uniform process, the experiment was carried out 3 times and the mean was calculated. A spectrophotometer was utilized to measure the removal and decolorization rates of the samples [30].

### GC/MS Analysis

The degraded by-products from the previous experiment were identified by GC/MS analysis. After 24 h of incubation, 50 mL of the culture supernatant was extracted with an equivalent volume of ethyl acetate. A rotary evaporator at 40 °C condensed the ethyl acetate extract to 3 mL volume. 3 μL of the condensed extract was injected into the GC/MS ISQ QD (Thermo Scientific) instrument. A TG-5 MS Zebron capillary column with a 30 m × 0.25 mm ID × 0.25 μm film thickness was used for the analysis. Helium was used as the mobile phase with a flow rate of 1 mL/min. Temperature in the oven was held at 50 °C for 6 min before increasing to 210 °C for 2 min (23 °C/min), then 210–305 °C for 14 min (30 °C/min). The injector temperature was 270 °C. The pattern of fragmentation in mass spectra was automatically linked with the NIST database mass library using GC–MS software (Thermo Xcalibur) [31].

### Toxicity test

Acute toxicity assays were carried out on *Artemia salina*, which was utilized as a bioindicator, to assess the effect of BB 41 and its metabolites on living organisms after biodegradation. In a flask, 1 g of *Artemia* cysts was cultured in 1 L of artificial seawater at 30 °C. An aquarium air pump and a fluorescence lamp were used to provide aeration and light illumination for the *Artemia* culture. Within 24 h of incubation, *A. salina* cysts begin to hatch, and the hatched larvae were placed in a fresh seawater medium for 24 and 48 h before being employed in a toxicity test [32].

The samples were taken before and after the biodegradation experiment, which used coated A1 to remove BB 41 dye at varied concentrations (100, 200, and 300 ppm). Ten milliliters of each BB 41 concentration was prepared and poured into 20-mL sterilized plastic plates. Artificial seawater was used for the untreated samples, 8 mL of the samples after treatment were diluted with 2 mL of artificial seawater to make a total of 10 mL of test solution, then 10 larvae were added and exposed to the solutions for 24 h to detect toxicity (mortality rate). Only seawater was used in the control sample [33]. The following equation was used to calculate the mortality rate.

$$\mathrm{Mortality \%}=\mathrm{Dead\;larvae}/\mathrm{total\;larvae}\times\;100$$

### Application of Coated A1 on Real Industrial Wastewater

To test the possibility of using the prepared coated A1 to remove color from a real wastewater sample, a sample of industrial wastewater was taken from the waste effluent of a textile and dyeing facility in Alexandria, Egypt. The sample was chosen to look into a UV–Vis spectrophotometer, scanning the sample in the range of 200 to 700 nm to measure dye removal capabilities.

Two dosages of coated A1 (0.5 mL and 1 mL) were inoculated in a 100 mL of effluent and incubated at 37 °C under static conditions for 96 h. The color removal % was determined using spectrophotometry on the supernatant after centrifugation [34].

The approach given in Standard Methods for the Examination of Water and Wastewater was followed to measure soluble COD. The percentage of COD reduction (CR %) was calculated as following:

$$\mathrm{CR \%}={\mathrm{COD}}_{0}-{\mathrm{COD}}_{\mathrm{t}}/{\mathrm{COD}}_{0}$$

where COD_0_ is the initial COD value (at 0 h) and COD_*t*_ is the observed COD value after a specific reaction time (*t*).

## Results and Discussion

### Isolation and Screening of Dye by Degrading Bacterial Isolate

**Fig. 1:**
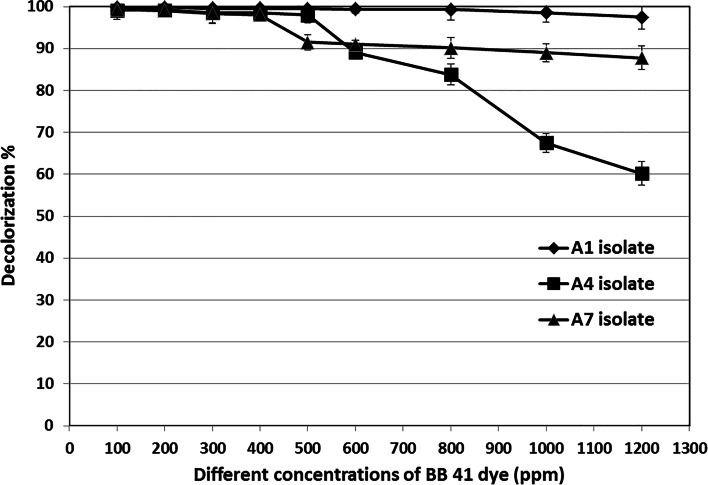
Effect of BB 41 dye concentration on decolorization % of different isolates

The soil and wastewater samples were gathered from various locations near the waste of some local textile industrial factories in Alexandria, Egypt. About 50 isolates were picked up that showed decolorization zone on agar plates containing BB 41 dye. Then, 7 isolates were selected for secondary screening, and they were named from A1 to A7. Three effective isolates, A1, A4, and A7, showed maximum decolorization ability of BB 41 dye (100–1200 ppm) which reached 97.49, 60.20, and 87.81% at 1200 ppm, respectively, after 48 h of incubation (Fig. 1). Lin et al. [35] found that some aerobic bacteria can use oxygen catalyzed azoreductase to degrade azo compounds and generate aromatic amines. These bacteria grow by reductively cleaving –N = N–bonds and using amines as a carbon and energy source.

### Molecular Identification of the Bacterial Isolate

A ribosomal RNA 16S gene sequence was used to confirm the identity of the bacterial isolate A1. The 16S rRNA sequence was determined to be 999 bp in length. It was examined using BLASTn, and multiple sequence alignment was done with CLUSTAL W. A phylogenetic tree was created by BioEdit [36] based on phenotypic characteristics and phylogenetic analyses. The isolate A1 was identified as *Raoultella Ornithinolytica* sp., and it was confirmed as 99% identical to *Raoultella Ornithinolytica* sp. strain DUCC3747 (KP318494) and deposited in the GenBank database under accession number KT213695.

### Optimization of A1 Growth Conditions for BB 41 Dye Decolorization

Strain A1 was characterized by the significant decolorizing activity and tolerance of BB 41 concentrations (up to 1600 ppm). Under static conditions, the optimum pH and temperature for maximum decolorization ability were reported to be 6–7 and 37 °C, respectively (data not shown).

### Characterization of the Prepared Coated A1

**Fig. 2:**
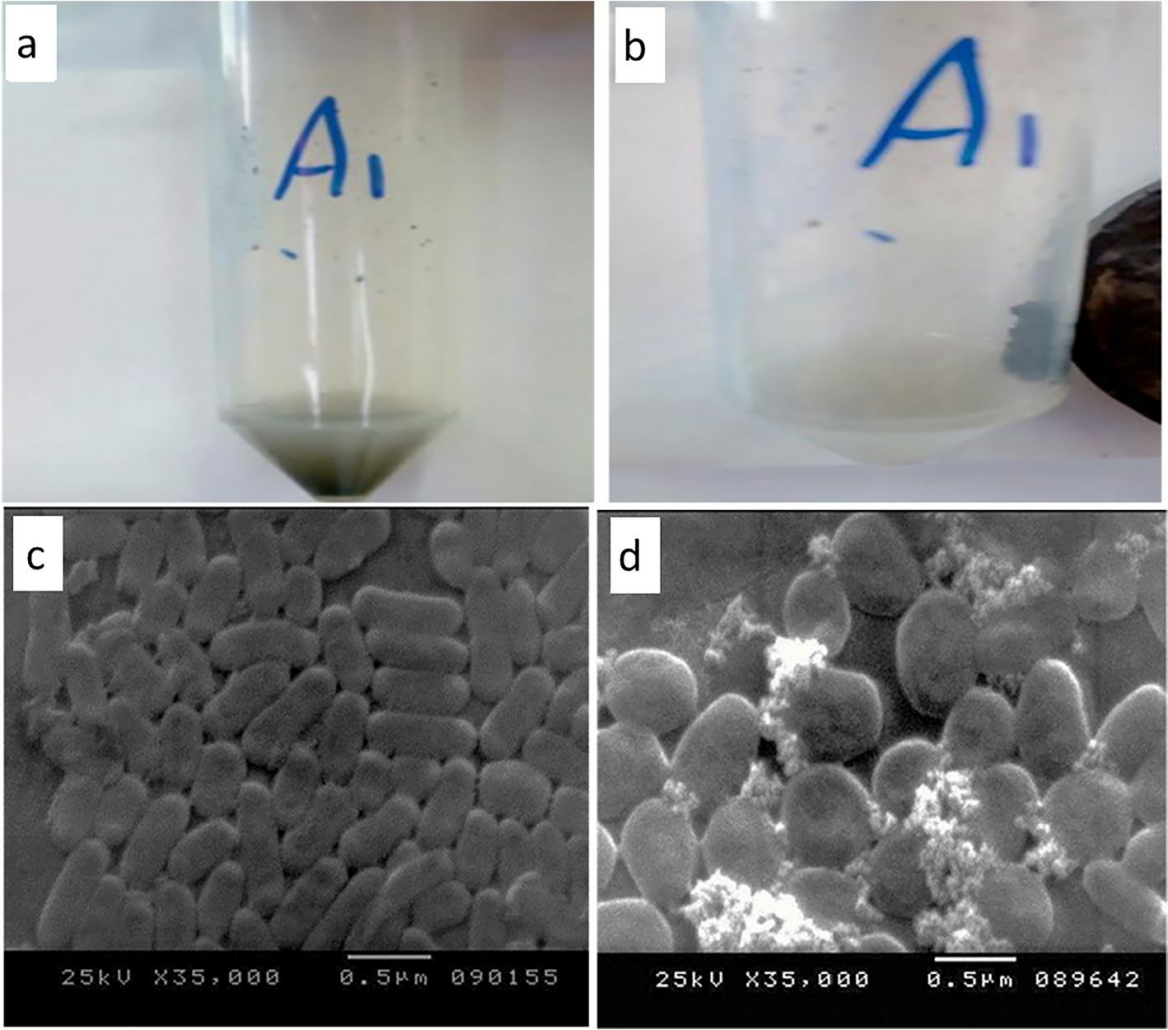
**a** Prepared coated A1 without external magnetic field. **b** Coated A1 with an external magnetic field. **c** SEM image of free A1. **d** SEM image of coated A1 with magnetic Fe_3_O_4_ NPs

The physiological conditions of bacterial cells and the physicochemical features of nanoparticles are thought to be the most important factors that influence nanoparticle-bacterial cell interactions. The surface charge of both nanoparticles and bacterial cell walls was considered as the most important interaction factor. Many investigations have shown that bacterial cells adhere to both naked and surface-modified Fe_3_O_4_ NPs primarily by electrostatic contact [37–39]. The theory that Fe_3_O_4_ NPs surface charge turns positive or negative depending on the pH of the solution helped to clarify the formulation of coated A1. Fe_3_O_4_ NPs have a positive surface charge in acidic medium (pH of LB broth is usually 5.6). Petrova et al. [40] and Attallah et al. [41] clarified this behavior, which is expected as the pH point zero of charge (PZC) value of Fe_3_O_4_ is around 6 (PZC is the pH value at which magnetite surface charge is close to neutral), so in an acidic medium, Fe_3_O_4_ NPs are positively charged. The gram-negative *Raoultella ornithinolytica* sp. A1 with a polyanionic cell membrane can combine with positively charged Fe_3_O_4_ NPs through adsorption mediated by electrostatic contact. Bacterial strain A1 grown to a late exponential phase was mixed with synthetic magnetic Fe_3_O_4_ NPs for 2 h to allow exponentially growing bacteria to interact with the NPs and complete the immobilization process. The coated cells in the liquid medium were separated by an external magnet after the NPs coated them through adsorption, as shown in Fig. 2a, b. The bacteria that were not coated (cells that were not attracted to the magnet) were then discarded.

Scanning electron microscopy was utilized to analyze the structural and morphological analyses of the produced coated bacterial cells with magnetic Fe_3_O_4_ NPs. As shown in Fig. 2c and d, SEM images revealed that bacterial cells were uniformly disseminated and coated with Fe_3_O_4_ NPs.

**Fig. 3:**
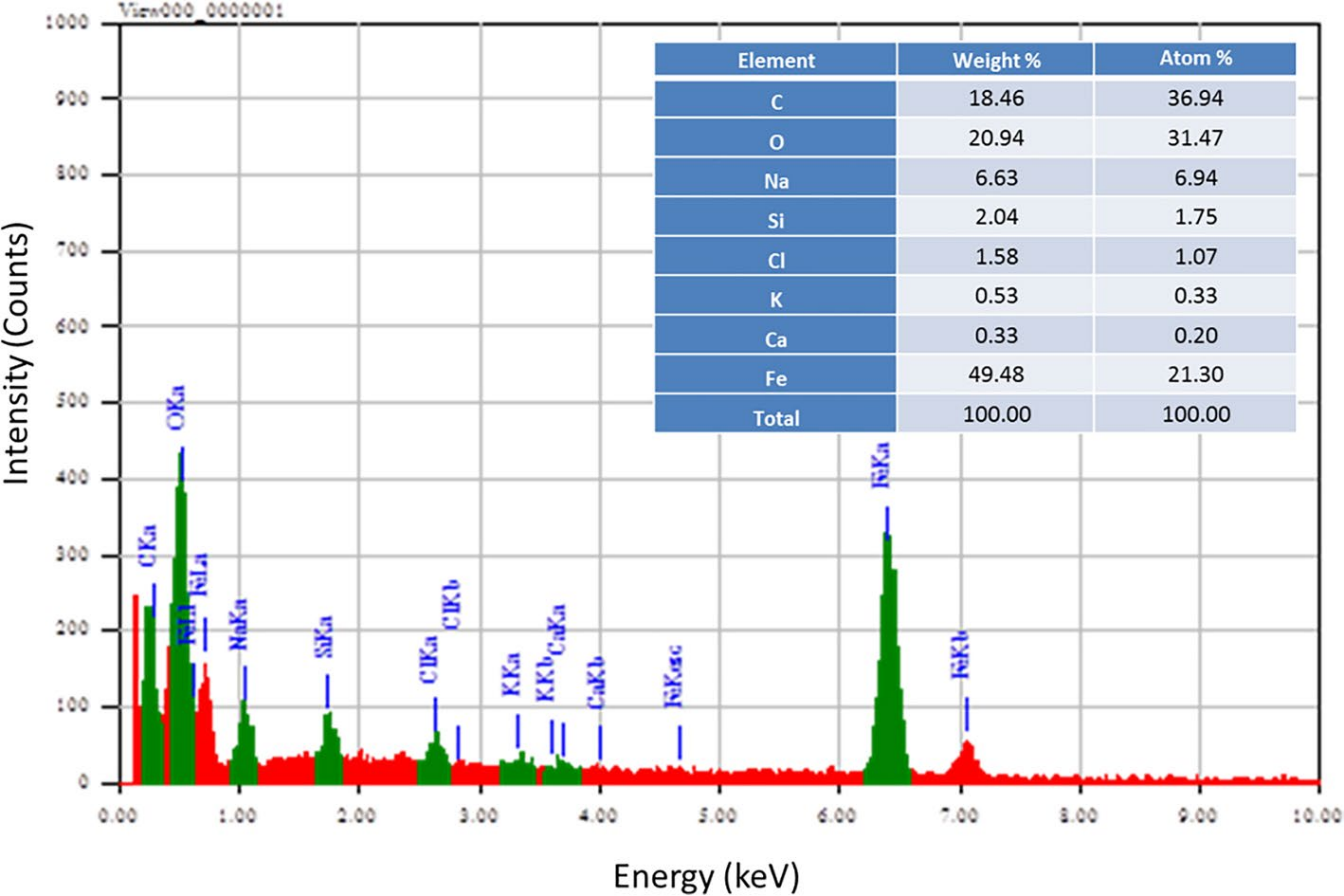
SEM–EDX spectrum shows the elemental composition of the coated A1

SEM–EDX was used to determine the elemental composition of immobilized bacteria A1 that had been coated with Fe_3_O_4_ NPs. SEM–EDX spectrum revealed intense peak signals of iron, carbon, oxygen, sodium, chloride, calcium, potassium, and silicon, as shown in Fig. 3. The existence of oxygen and iron signals was attributed to the Fe_3_O_4_ NPs combination. The carbon signal was detected mainly due to the existence of carbon-containing molecules in bacterial cells. Other signal peaks were traces; Na and CL from impurities during Fe_3_O_4_ NPs synthesis. Other elements like Ca and K may be impurities created from bacterial nutrients. The glass grid on which the sample was sited through the examination caused the silicon peak signal. These results validated the formation of coated *Raoultella Ornithinolytica* sp. A1 (coated A1).

### Application of Prepared Coated A1 with Fe_3_O_4_ NPs in BB 41 Dye Decolorization

**Fig. 4:**
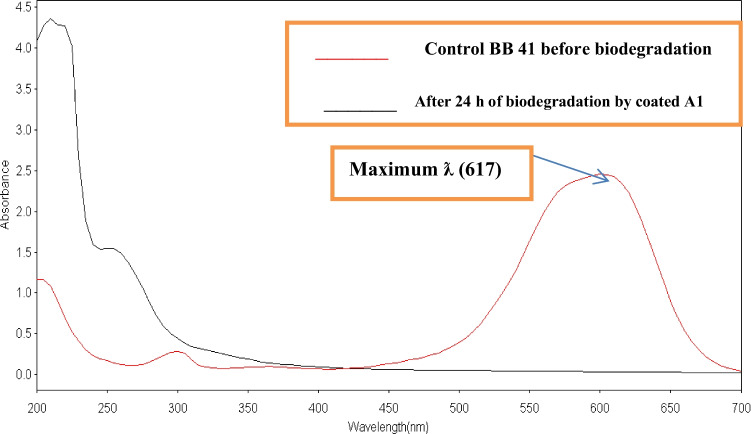
UV–Vis spectrum of Basic Blue 41 (200 ppm) before and after biodegradation treatment with coated A1

The results of UV–Vis examinations were carried out in the 200–700 nm range, as shown in Fig. 4, which revealed that after 24 h, the decolorization of 200 ppm BB 41 dye utilizing coated A1 was 100%. During microbiological treatment, the UV–Vis spectrum showed a considerable spectral change in the dye molecules, with a shift in the dye’s maximum absorbance (617 nm) and the peak fading away, indicating that BB 41 azo dye decolorization occurs by bacterial biodegradation. According to the findings, Fe_3_O_4_ NPs may increase bacterial bioactivity and metabolite production due to their larger specific surface area and greater surface energy, hence producing more available active sites. Furthermore, the study showed that Fe_3_O_4_ NPs enhanced bacteria’s growth, metabolism, and enzyme activities (i.e., iron or iron ions released from Fe_3_O_4_ NPs can be used as a source of iron and increase cell growth) [30].

### Influence of Different Dye Concentrations

**Fig. 5:**
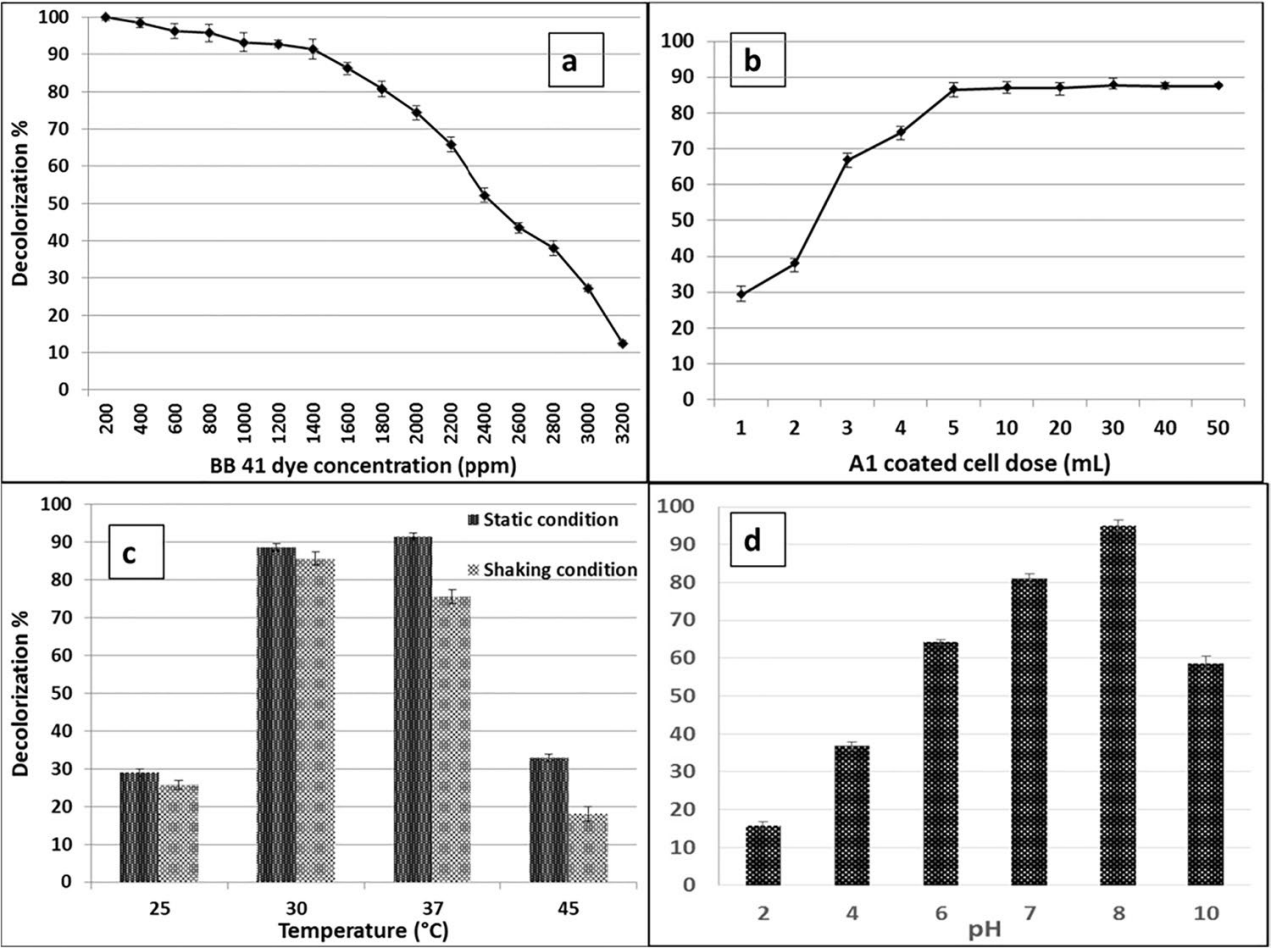
Decolorization percentage of BB 41 dye after 24 h under different conditions. **a** Effect of different BB 41 dye concentrations by using coated A1 at 30 °C. **b** Effect of coated A1 dose with dye concentration 1600 ppm at 30 °C. **c** Effect of temperature and static or shaking conditions with dye concentration 1600 ppm by using 5 mL/L coated A1. **d** Effect of different pH of culture medium with dye concentration 1600 ppm using 5 mL/L coated A1 at 37 °C

The effect of initial BB 41 dye concentration (200 to 3200 ppm) on decolorization percentage was investigated using coated *Raoultella ornithinolytica* sp. A1 cells, which were cultured at 30 °C for 24 h and 200 rpm shaking conditions. At 1400 ppm, the decolorization percentage of BB 41 dye is considerable, reaching 91.36%. The removal percentage remained nearly constant until 2000 ppm, then declined to 65.82% at 2200 ppm, and then continued to decrease as the BB 41 concentration increased, reaching 12.53% at 3200 ppm, as shown in Fig. 5a. These findings indicated that even at high initial concentrations of dyestuff, coated A1 had a high removal and decolorizing performance. According to Khehra et al. [42] and Kalme et al. [43], dye decolorization can be severely impeded at high dye concentrations due to the dye’s toxic effect on degrading microorganisms. This could be explained by the insufficient bacterial biomass concentration and blockage of active sites of azoreductase by dye molecules with different structures.

At a greater initial dye concentration, reactive group azo dyes with sulfonic (SO_3_H) groups on their aromatic rings significantly reduced the growth of microorganisms, as did BB 41 azo dye with (SO_3_H) groups [44].

Similarly, Ali et al. [45] used innovative bio-magnetic membrane capsules (BMMCs) of 0.02 g/L dose at pH 6.5 against different concentrations of malachite green (MG) dye. The removal effectiveness of MG dye declined from 98.61 to 1.5% when the initial MG dye concentration was increased from 25 to 1000 mg/L.

### Influence of Coated Bacterial Cell Dose

The influence of coated *Raoultella ornithinolytica* sp. A1 dose on the decolorization % of BB 41 azo dye was studied using (1–50 mL/L bacterial dosage) with an initial concentration 1600 ppm of BB 41 and incubated at 30 °C for 24 h under 200 rpm shaking. When the dose of the coated A1 cell was increased from 1 to 5 mL/L, the effectiveness of the coated A1 cell in decolorization percentage increased from 29.07 to 86.69%, as shown in Fig. 5b. The percentage of decolorization improved slightly when the dose was increased until it reached a maximum of 87.69% at 30 mL/L bacterial dose. The decolorization efficiency increased steadily as the coated A1 dose increased to 50 mL/L, reaching 87.44% after 24 h. There was no great difference by using 5 or 50 mL/L of coated A1 in dye decolorization, so bacterial dosage of 5 mL/L was selected as an optimum dosage for a commercial reason and to reduce materials usage on a large scale. The increase in the active sites of the coated cells with magnetic Fe_3_O_4_ NPs available for dye molecules could explain the progress shown in the removal % and decolorization of azo dyes. However, the increase in dye removal was not linear. The aggregation of magnetic Fe_3_O_4_ NPs that coated the bacterial cell with dose increment from 30 to 50 mL/L could explain this, by reducing the total active sites of the bacterial cell mass coated with Fe_3_O_4_ NPs.

Junnarkar et al. [46] also reported employing unique bacterial consortia to decolorize Direct Red 81 (DR 81). The decolorizing ability was tested at different inoculum concentrations ranging from 5 to 30% (v/v) at pH 7.0 and a 37 °C incubation temperature. The results showed that the rate of decolorization increased as the inoculum size rose, up to 20% of the inoculum size. However, the rate of decolorization did not differ significantly after 20% (v/v) inoculum size.

These data are in agreement with Ahmed et al. [47]. They employed varied doses from 0.01 to 1 g of WFS coated with Mg/Fe-LDHs nanoparticles (magnesium/iron (Mg/Fe)-layered double hydroxides (LDHs) nanoparticles immobilized on waste foundry sand) for Congo red sorption. Their research showed that increasing sorbent dose improved removal performance for a constant dye concentration due to the fact that the higher the sorbent dosage in a solution, the more active sites are available.

### Influence of Temperature and Static or Shaking Conditions

The efficiency of coated *Raoultella ornithinolytica* sp. A1 for BB 41 azo dye decolorization was investigated under various temperatures/static or shaking conditions. After 24 h, the percentage of decolorization was determined using different incubation temperatures (25, 30, 37, and 45 °C) under static and shaking (200 rpm) conditions with an initial concentration of 1600 ppm of BB 41 dye and 5 mL of coated A1/L dye. The decolorization efficiency reached 91.43% at 37 °C incubation temperature and static conditions, and 75.59% at 37 °C incubation temperature and shaking conditions (200 rpm), as shown in Fig. 5c. According to these results, the optimum temperature for BB 41 dye decolorization by coated A1 is 37 °C at static conditions. Shah et al. [48] demonstrated that high temperatures cause thermal inactivation of proteins, which can be related to the loss of cell viability or denaturation of the azoreductase enzyme. Furthermore, according to Solís et al. [49], the presence or absence of oxygen has a significant effect on the biodegradation of azo dye. Thus, decolorization of azo dyes may be enhanced or inhibited by aeration and agitation. Our findings were consistent with those of EMMS et al. [50], who discovered that native bacterial strains (*Alcaligenes faecalis*, *Micrococcus luteus*, and *Staphylococcus warneri*) completely decolorized CI Direct Blue 201 textile dye after 60, 64, and 72 h of incubation time, under static conditions at 28 °C. Also, Shah et al. [26] discovered that utilizing *Bacillus* ETL-1982 strain, the decolorization of Reactive Red azo dye with an initial dye concentration of 0.5 g/L was 85–95% within 24 h under static conditions, compared to 35% under shaking conditions.

### Influence of Culture Medium pH

The influence of LB culture medium pH on coated A1 efficiency for the decolorization of BB 41 dye was examined in a range of 2, 4, 6, 7, 8, and 10 with an initial concentration of 1600 ppm under static conditions at 37 °C using 5 mL/L of coated A1 after 24 h. The decolorization effectiveness of dye was optimum at pH 8 with 94.90%, as illustrated in Fig. 5d. At pH 7 and 10, a moderate level of dye decolorization was produced at 81.04% and 58.61%, respectively. However, the removal and decolorization of BB 41 dye were shown to be inhibited at pH 2 and 4. Chang et al. [51] found that the high rate of degradation at optimal pH is linked to the transfer of dye molecules through the cell membrane, which is the rate-limiting stage in degradation. Changes in pH can influence not just the shape of an enzyme, but also the shape or charge characteristics of the substrate, which prevents the substrate from binding to the active site or undergoing catalysis, according to Etemadifar et al. [52]. Rajeswari et al. [53] discovered that the maximal removal of reactive dyes by employing *Lysinibacillus sphaericus* RSV-1 was 94.37% and 91.99% at pH 7 and 9, respectively, after 36 h of incubation. But when live bacterial cells are used, neutral or slightly alkaline pH values are frequently the best for color removal.

### Influence of Incubation Time

The influence of incubation time on the effectiveness of coated A1 for decolorization of BB 41 was studied from 3 to 24 h in static conditions at 37 °C using 5 mL of coated A1/L dye with an initial concentration of 1600 ppm and a pH of 8. The results showed that the decolorization efficacy improved with time, with a minor change in decolorization after 3 h to 20.25%, then increased sharply to reach equilibrium time after 18 h with 88.91% of decolorization, and then increased slowly until 95.22% after 24 h. These findings show that the BB 41 elimination process occurs in two stages. The first acclimation step requires around 6 h of incubation for early adaption of the bacterial biomass in the current dye environment for protein content screening (azoreductase enzyme which is responsible for dye biodegradation and decolorization) [54]. The dye uptake in the second stage is rapid, reaching equilibrium after 18 h of incubation. According to Dellamatrice et al. [55], acclimation is necessary for dye decolorization. Similar to our result, Siddeeg et al. [56] established that MB and RO 16 dyes decolorization rates increased using MnP/Fe_3_O_4_/chitosan nanocomposite with an increase in incubation period from 10 to 50 min to reach 96% ± 2% and 98% ± 2%, respectively.

### Continuous Batch Cycle Process

The ability of the coated *Raoultella ornithinolytica* sp. A1 for the decolorization of repeated additions of BB 41 dye concentration (1600 ppm) for each cycle was tested using continuous batch cycles decolorization and degradation. The experiment was done under ideal conditions for dye decolorization by coated A1 (LB broth medium pH 8, 37 °C, static conditions, and 5 mL/L coated A1 dosage). The coated A1 was used for decolorization four times (every 24 h) with high efficiency, reaching 95.27, 93.66, 90.51, and 80.14%, respectively. Based on these findings, we hypothesized that *Raoultella ornithinolytica* sp. A1 coated with magnetic Fe_3_O_4_ NPs facilitates the re-use of bacteria via magnetic separation and maintains high catalytic activity for multiple degradation cycles. Additionally, Liu et al. [57] discovered that adding Fe_3_O_4_ nanoparticles increased the decolorization activity of immobilized cells because Fe_3_O_4_ NPs have the ability to promote microbial extracellular electron transfer to electron acceptors. Moreover, Nadi et al. [58] investigated the removal of Congo Red dye by *Bacillus subtilis* cells immobilized by iron oxide nanoparticles, showing that the immobilized bacteria were effectively recycled for up to seven decolorization cycles, with a removal rate of more than 80% at various pH values.

### Analysis of Metabolites During Biodegradation of BB 41

**Fig. 6:**
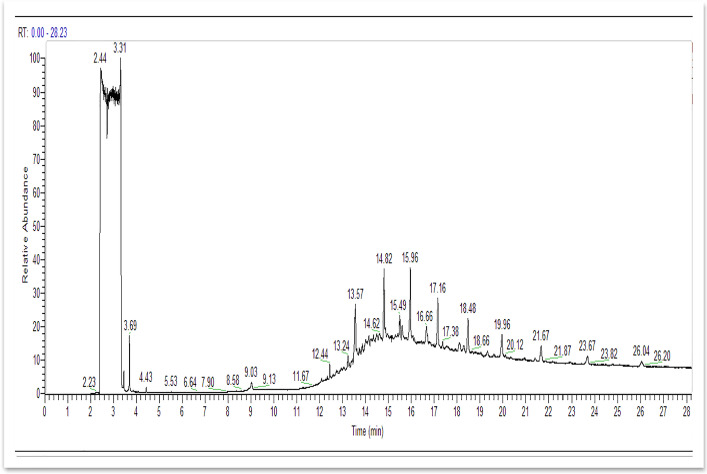
GC-mass spectra of identified metabolites after biodegradation of BB 41 azo dye by coated *Raoultella ornithinolytica* sp. A1 after 24 h

GC–MS analysis was used to identify the biodegradation intermediates of the treated BB 41 azo dye samples utilizing coated A1. The components were identified by comparing their spectra to those of reference substances in a mass library (NIST database). Several peaks were identified; as shown in Table 1 and Fig. 6, their outputs were reflected as two primary peaks with retention times 2.44 min and 3.31 min showing a maximum relative abundance in the GC chromatogram. Few peaks were also identified at 2.67 min, 2.75 min, 2.87 min, 2.92 min, 3.45 min, 3.7 min, 13.05 min, and 15.5 min matching the library database; all outputs were perceived after 24 h of dye degradation. This result proposes that under aerobic conditions, coated *Raoultella ornithinolytica* sp. A1 reductively cleaves the azo bond (N = N) with the help of an azoreductase enzyme, resulting in intermediate metabolites (e.g., aromatic amines), and further degradation resulted in more ionized compounds such as the benzene ring indicating full degradation was achieved. These findings indicate that mono- and di-oxygenase enzymes catalyze the integration of oxygen from O_2_ into the aromatic ring of organic molecules former to ring fission in aerobic conditions. With the help of oxygen-catalyzed azoreductases, some aerobic bacteria may reduce azo compounds and generate aromatic amines. It was also discovered that the aerobic azoreductase could reductively cleave both carboxylated and sulfonated structural analogues. According to Solís et al. [49] and Saratale et al. [59], only a few bacteria are capable of growing solely on azo chemicals. Using azo dye-reducing enzymes that have been identified to degrade azo dyes under aerobic conditions, these bacteria cleave –N = N– bonds reductively and use amines as an energy source for their growth.

**Table 1:**
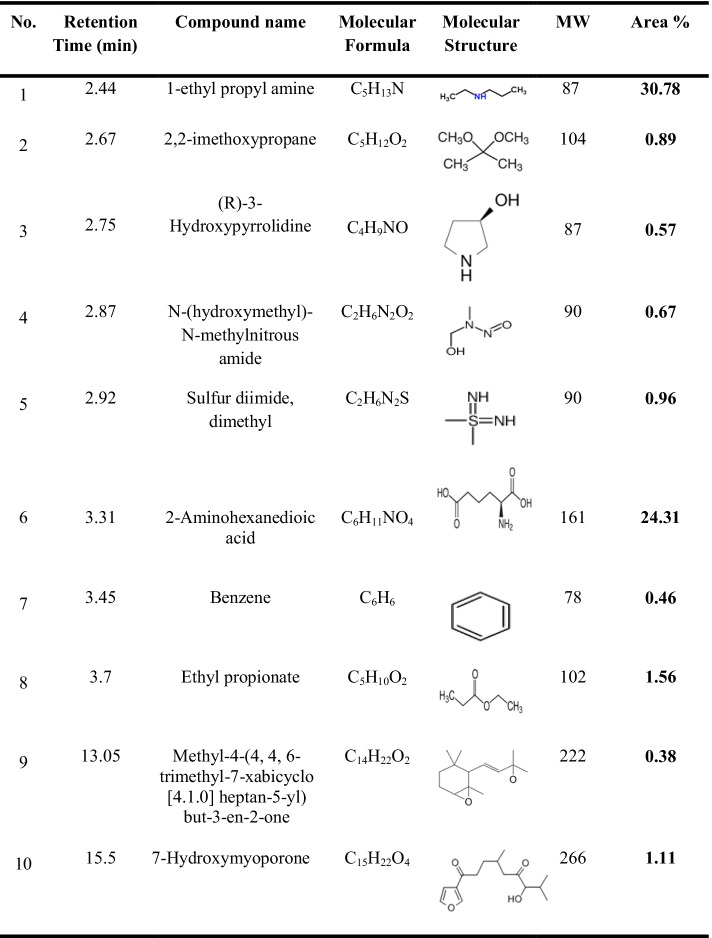
Identified metabolites after biodegradation of BB 41 dye by coated *Raoultella ornithinolytica* sp. A1 using GC–MS

### Toxicity Test

**Fig. 7:**
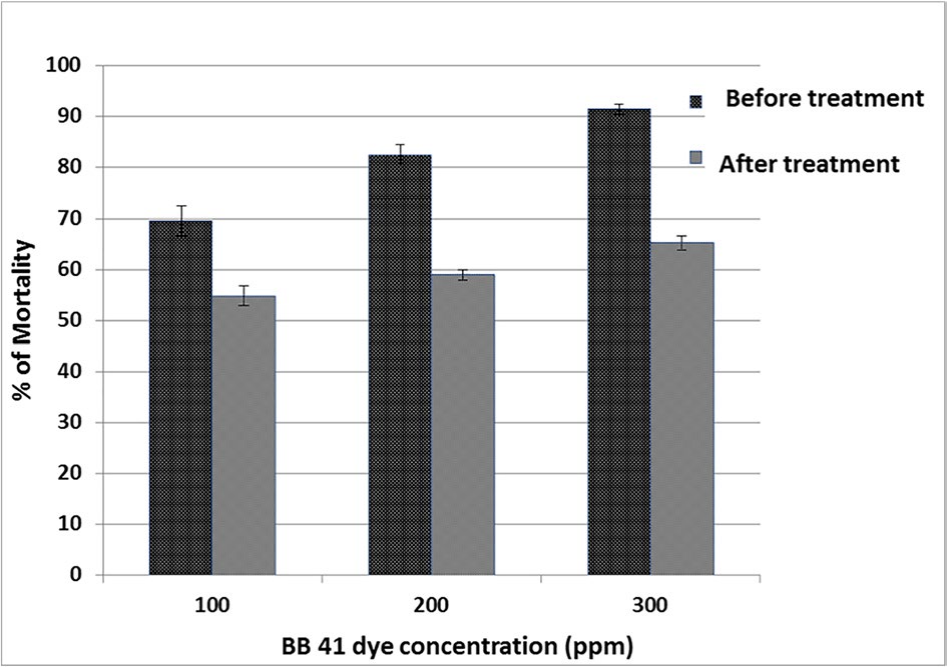
Acute toxicity test for BB 41 dye before and after treatment by using *Artemia salina* after 24 h

The toxicity test was carried out to confirm the treatment’s efficiency and to assess the environmental toxicity of biodegradation products. *Artemia salina* (brine shrimp) 48 h larvae were used in a 24 h acute toxicity test. Before and after biodegradation treatment by coated A1 at their optimum conditions, decolorized samples of BB 41 azo dye with initial concentrations (100, 200, and 300 ppm) were prepared. The mortality rate of *A. salina* was 69.55, 82.42, and 91.27%, before treatment with 100, 200, and 300 ppm BB 41 dye, respectively. The mortality rate was reduced after treatment for the same dye concentrations 54.76, 58.91, and 65.20%, respectively, as indicated in Fig. 7. We conclude from our results that BB 41 dye is poisonous to *Artemia salina* as a bio-indicator test organism before treatment. BB 41 dye biodegradation by coated A1 was effective in lowering the harmful result, indicating a reduction of generated hazardous intermediates or its transformation into an ecologically neutral final product. BB 41 azo dye toxicity was also confirmed; this may be due to the presence of a sulphanilic acid group (SO_3_H), which could resist biodegradation or hinder it partially degraded [44].

Swarnkumar et al. [60] proved that *Artemia salina* is an effective model for testing the aquatic toxicity of textile dyes and dye-containing effluents. In a study on reactive dyes and textile effluents, he used *Artemia salina* to calculate LC_50_ values and found the most poisonous dye is Reactive Orange with 1.7 µg/mL, while the effluent of textile processing industries (DU1) had an LC_50_ of 15%.

Similarly, Khouloud [61] found a mild toxicity impact with the highest dye metabolites after treating Congo Red and Methyl Red with *Lysobacter* sp. T312D9 at initial doses of 2000 and 1500 μg/mL utilizing *Artemia salina*. After 24 h, the LC_50_ for Methyl Red and Congo Red was determined to be 3.3 and 3.2, respectively. This means there were less harmful metabolites produced throughout the decolorization process.

### Application of Coated A1 on Real Industrial Wastewater

**Fig. 8:**
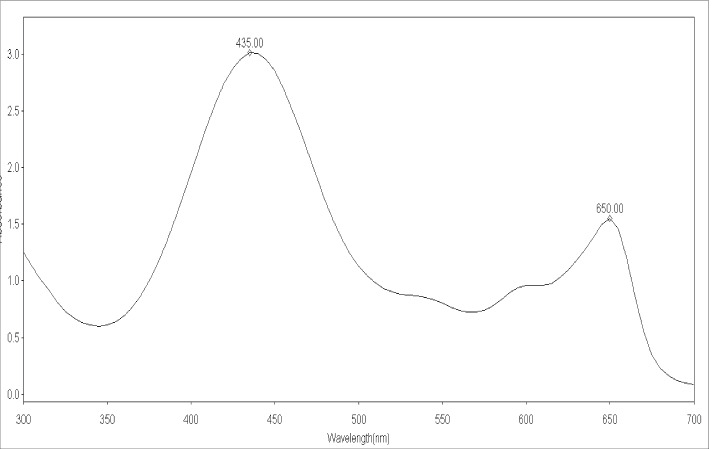
UV–visible scanning of a real dyeing wastewater sample

**Fig. 9:**
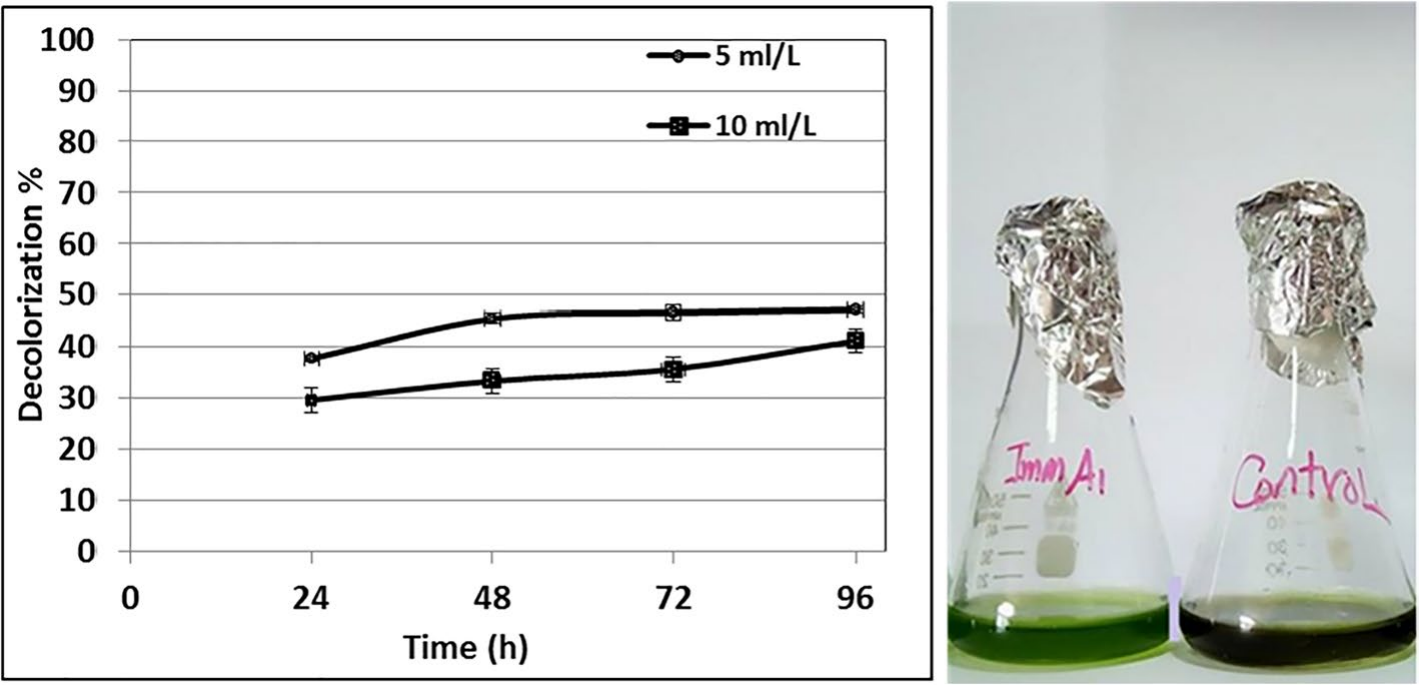
Decolorization percentage of colored wastewater sample by using different dosages of coated *Raoultella ornithinolytica* sp. A1

The potential application of coated *Raoultella ornithinolytica* sp. A1 for the elimination of color from a real wastewater sample was studied at optimum conditions (LB broth medium, pH 8, temp. 37 °C, static conditions). This experiment was performed using different doses (5 and 10 mL/L) of coated A1. Spectrophotometric scanning of the colored wastewater sample revealed the appearance of a peak at 435 nm, which corresponded to the maximum wavelength of a combination of dyes in the wastewater sample as shown in Fig. 8. The color of the wastewater sample was measured before and after treatments, with aliquots obtained from the solution at different time intervals (24, 48, 72, and 96 h). As shown in Fig. 9, treating colored wastewater samples with 5 mL dosage/L resulted in decolorization percentages of 37.70, 45.52, 46.61, and 47.20%, respectively. The percentages of decolorization were 29.62, 33.34, 35.67, and 41.18%, respectively, at 10 mL/L dosage. COD decreased from 592 to 494 ppm after 96 h of incubation when a 5 mL/L dosage was used.

Likewise, Fetyan et al. [62] stated that under static conditions at pH 7.0 and 37 °C, the color of textile effluent was completely removed within 8 h by utilizing immobilized cells (BMC) chitosan-magnetite nanoparticles. Our trial established that the higher decolorization and biodegradation efficiency of the treatment was achieved using a low dose of coated A1. That is due to the aggregation of magnetic Fe_3_O_4_ NPs that coat the bacterial cell upon increasing their mass from 5 to 10 mL/L, which in turn decreases the number of active sites of both the bacterial cell mass and Fe_3_O_4_ NPs.

## Conclusion

Aiming to improve dye removal and degradation, Fe_3_O_4_ NPs were used to immobilize bacterial cells; *Raoultella ornithinolytica* sp. A1 was isolated from contaminated local industrialized outflows in Alexandria, Egypt, and used in this study to measure dye removal capabilities. Bacterial coating with Fe_3_O_4_ NPs has emerged as a viable frame material for generating functional materials with a variety of surface functionalities, including degradation, adsorption, and magnetism collection for reuse. Using coated bacterial cells with magnetic Fe_3_O_4_, several factors have been evaluated to optimize BB 41 dye biodegradation. The capacity of coated bacteria to decolorize BB 41 azo dye was maximum (95.22%) when tested for 24 h under the following conditions: pH 8, 37 °C, static conditions, and 5 mL/L coated A1. Four consecutive batch cycles of dye removal using the coated bacteria resulted in a decolorization efficiency of 80.14%. Coated A1 outperformed free microorganisms in terms of biodegradation and recovery through reuse procedures. Furthermore, as compared to the untreated BB 41 dye samples, the decolorized BB41 dye water samples treated by coated A1 cells had no toxic effect on *Artemia salina*. We concluded from our investigation that eliminating color from actual wastewater samples using Fe_3_O_4_ NPs coated *Raoultella ornithinolytica* sp. was a simple, low-cost, and successful technique. Therefore, coating *Raoultella ornithinolytica* sp. A1 could be considered a promising strategy for enhancing wastewater biodegradation in the textile sector.

## Data Availability

Not applicable.
